# Targeted inhibition of cell-surface serine protease Hepsin blocks prostate cancer bone metastasis

**DOI:** 10.18632/oncotarget.1817

**Published:** 2014-03-16

**Authors:** Xi Tang, Sumit S. Mahajan, Liem T. Nguyen, François Béliveau, Richard Leduc, Julian A. Simon, Valeri Vasioukhin

**Affiliations:** ^1^ Division of Human Biology, Fred Hutchinson Cancer Research Center, Seattle, WA, USA; ^2^ Clinical Research Division, Fred Hutchinson Cancer Research Center, Seattle, WA, USA; ^3^ Department of Pharmacology, Université de Sherbrooke, Sherbrooke, Québec, Canada

**Keywords:** cancer, prostate cancer, metastasis, bone metastasis, protease, Hepsin

## Abstract

The development of effective therapies inhibiting prostate cancer progression and metastasis may substantially impact prostate cancer mortality and potentially reduce the rates of invasive treatments by enhancing the safety of active surveillance strategies. Hepsin (*HPN*) is a cell surface serine protease amplified in a subset of human sarcomas (7.2%), as well as in ovarian (10%), lung adeno (5.4%), lung squamous cell (4.5%), adenoid cystic (5%), breast (2.6%), uterine (1.7%) and colon (1.4%) carcinomas. While *HPN* is not amplified in prostate cancer, it is one of the most prominently overexpressed genes in the majority of human prostate tumors and genetic experiments in mice indicate that Hepsin promotes prostate cancer metastasis, particularly metastasis to the bone marrow. We report here the development, analysis and animal trial of the small-molecule Hepsin inhibitor HepIn-13. Long-term exposure to HepIn-13 inhibited bone, liver and lung metastasis in a murine model of metastatic prostate cancer. These findings indicate that inhibition of Hepsin with small-molecule compounds could provide an effective tool for attenuation of prostate cancer progression and metastasis.

## INTRODUCTION

Prostate cancer is the most common nonskin cancer in American males [1]. While organ-confined prostate tumors are usually curable, metastatic prostate cancer is highly resistant to therapeutic intervention and almost uniformly fatal. Therefore, the development of effective novel targeted therapies to inhibit prostate cancer metastasis could have a considerable impact on prostate cancer mortality.

*Hepsin* (*HPN*) is one of the most upregulated genes in human prostate cancer and is overexpressed in up to 90% of prostate tumors with levels often increased >10 fold [2-4]. Hepsin increases early in prostate cancer initiation and its high levels are maintained throughout progression and metastasis [3-6]. Hepsin belongs to the protein family of type-II transmembrane cell surface serine proteases [7, 8]. Hepsin can cleave and activate pro-uPA, pro-HGF, Laminin332 and pro-MSP [9-11]. Previous studies indicate that Hepsin overexpression plays an important role in prostate and ovarian cancer [12-15]. The levels of Hepsin expression correlate with the high Gleason score and are indicative of poor clinical outcome and relapse after prostatectomy [3-6]. Hepsin is not overexpressed in prostate cancer cell lines and overexpression of Hepsin in cells in culture results in cell detachment and downregulation of cell proliferation [16, 17]. Hepsin upregulation in the context of the prostate gland *in vivo* promotes SV40 large T antigen-driven prostate cancer progression and metastasis to the liver, lung and bone [12]. Furthermore, Hepsin overexpression in the LNCaP human prostate cancer cell line grown as an orthotopic xenograft in mice promotes invasive tumor growth and lymph node metastasis [18].

In this study we report the development of a novel, non-toxic, and orally bioavailable small molecule Hepsin inhibitor, HepIn-13. We show that long-term exposure to HepIn-13 blocks prostate cancer metastasis in a preclinical genetic model of metastatic prostate cancer.

## RESULTS

### Identification of novel small molecule Hepsin inhibitors

Hepsin is prominently overexpressed in the majority of human prostate cancers and functional *in vivo* studies support a causal role for Hepsin in cancer progression [12, 18, 19]. Interestingly, while most of the cancer literature is primarily focused on Hepsin in prostate cancer, analysis of publically available datasets indicates that *Hepsin* is frequently amplified in a variety of human cancer types, especially in ovarian serous adenocarcinoma (10%), sarcoma (7.2%), lung adenocarcinoma (5.4%), lung squamous cell carcinoma (4.5%), adenoid cystic carcinoma (5%), breast carcinoma (2.6%), as well as many other cancer types (Figure S1). We hypothesized that inhibition of Hepsin activity using small molecules would attenuate prostate cancer progression and may have therapeutic potential in other cancers with *Hepsin* amplification.

**Figure 1:**
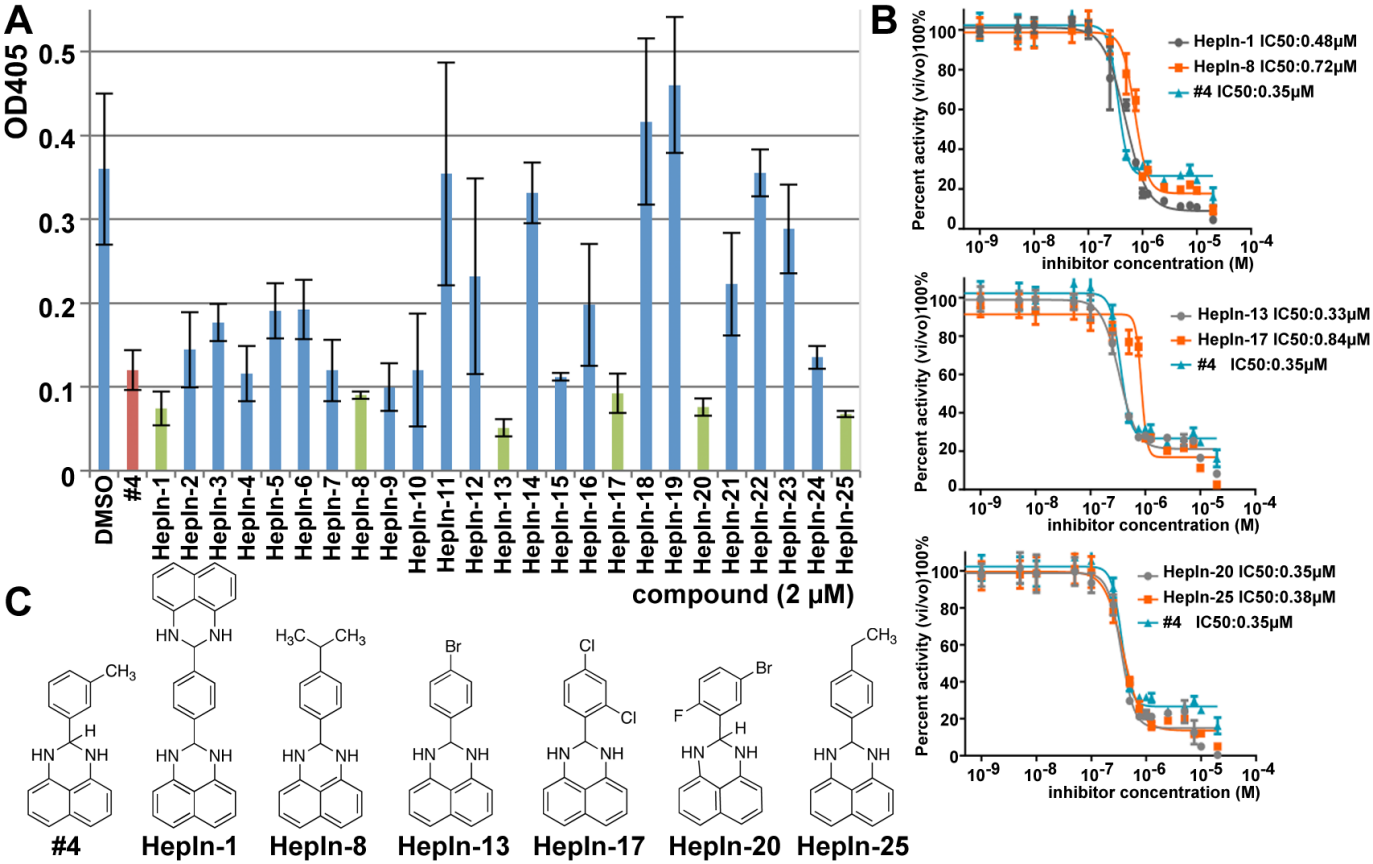
Identification of novel small molecule Hepsin inhibitors (A) Attenuation of Hepsin-dependent proteolytic activity by the lead compound #4 [20] and its derivatives. Purified recombinant Hepsin was preincubated with 2 μM of the indicated compounds for 30 min. The residual percent activity of the enzyme toward the chromogenic substrate was determined using a microplate reader at 405 nm. Data are the means of three independent experiments ±SD. (B) IC50 determination for Hepsin inhibitors #4, HepIn-1, HepIn-8, HepIn-13, HepIn-17, HepIn-20, HepIn-25. Data are the means of three independent experiments ±SD. (C) Chemical structures of identified Hepsin inhibitors.

We have previously identified several small molecule compounds that inhibit the activity of purified recombinant Hepsin [20]. To develop and analyze therapeutically-relevant Hepsin inhibitor, we analyzed all available from ChemBridge derivatives of the lead compound #4 (Figure 1). In these studies we used recombinant human Hepsin produced in Drosophila S2 cells [21] (Figure S2). While the majority of these compounds either did not show inhibition or inhibited Hepsin with decreased potency, six compounds (HepIn-1, HepIn-8, HepIn-13, HepIn-17, HepIn-20 and HepIn-25) displayed similar or increased potency (Figure 1, A-B). IC_50_ values were determined by titration against Hepsin activity and HepIn-13 was found to be the most potent inhibitor with an IC_50_ of 0.33 μM. (Figure 1, B). Similarly to compound #4, the identified derivatives were specific for Hepsin, as they showed only minor activity against Matriptase, a serine protease highly similar to Hepsin (Figure S3).

Since our Hepsin activity assay utilizes a small peptide substrate, it was necessary to analyze whether the identified compounds inhibit Hepsin-mediated cleavage of a protein substrate. It has been previously reported that Hepsin can cleave and activate pro-HGF [10, 11]. This Hepsin activity is likely to be important for prostate cancer progression, because HGF/MET signaling pathway is strongly implicated in tumor progression and metastasis in prostate cancer [22]. Thus, we analyzed whether our compounds can inhibit Hepsin-mediated cleavage of pro-HGF. We found that both the original lead compound #4 and its six derivatives inhibited Hepsin-mediated cleavage of pro-HGF (Figure S4, A-B). Therefore, we conclude that we identified several novel small molecule inhibitors, which inhibit the *in vitro* activity of recombinant Hepsin at sub-micromolar concentrations.

### Inhibition of Cell Surface Hepsin proteolytic activity

**Figure 2:**
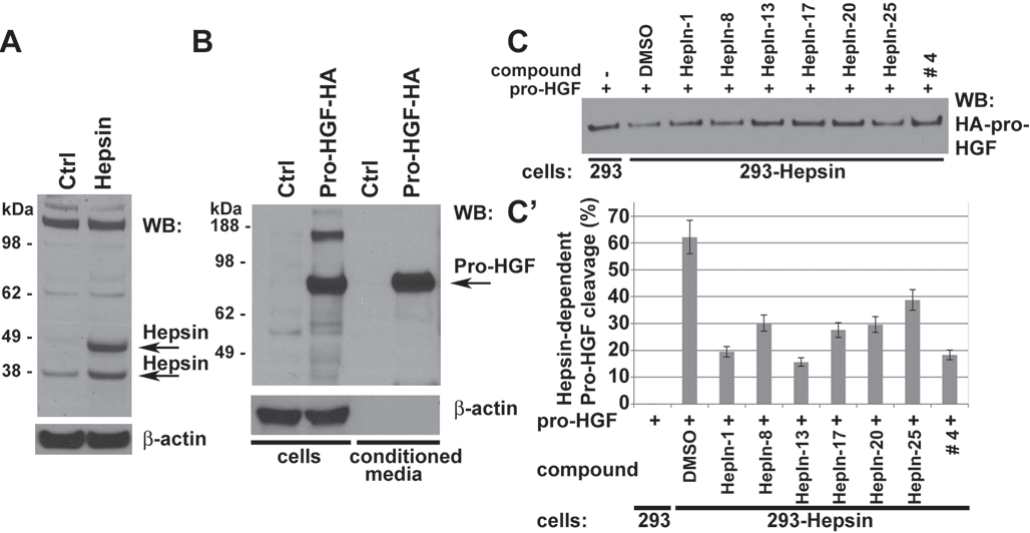
Hepsin inhibitors attenuate the Hepsin-mediated cleavage of pro-HGF in cell based activity assays (A) Western blot (WB) analysis of vector control- (Ctrl) or Hepsin-expressing HEK 293 cells with anti-Hepsin and anti-β-actin antibodies. Note that Hepsin is present as both full-length (precursor) and cleaved (activated) enzymes. (B) Western blot (WB) analysis of vector control (Ctrl) or pro-HGF-HA expressing HEK 293 cells and their conditioned media with anti-HA and anti-β-actin antibodies. (C) Cell-based Hepsin activity assay. Control or Hepsin-expressing 293 cells in the presence of DMSO or 10μM of indicated compounds were cultured for 2 hours in media containing pro-HGF-HA, and the remaining uncleaved pro-HGF-HA was detected by Western blotting with anti-HA antibodies. (C') Quantitation of data shown in C. Data are the means of three independent experiments ±SD

To determine whether the identified compounds can suppress the activity of full-length Hepsin, when it is expressed on the surface of live cells, we developed a cell-based Hepsin activity assay. For this purpose, we generated HEK293 cells overexpressing full-length Hepsin (Figure 2, A). HA-tagged human pro-HGF secreted into serum-free conditioned media from stably transduced HEK293 cells was used as a protein substrate in these experiments (Figure 2, B). Hepsin overexpressing, but not the control vector-transduced cells, efficiently cleaved the HA-tagged pro-HGF (Figure 2, C). This cleavage was inhibited in the presence of Hepsin inhibitors (Figure 2, C-C').

### Pharmacokinetics and oral bioavailability of Hepsin inhibitors

To determine whether the Hepsin inhibitors were suitable for in vivo use, we analyzed them in mice. Intravenous injections of escalating amounts of the compounds did not result in any signs of acute toxicity, even when injected at the maximal practical dose of a 20 mg/kg.

**Figure 3:**
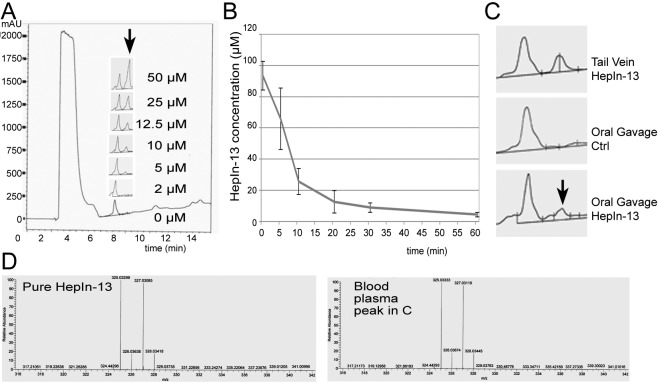
Pharmacokinetics and oral bioavailability of the Hepsin inhibitor HepIn-13 (A) Analytical HPLC profiles (mAU at 220 nm) of HepIn-13 which was dissolved in the mouse blood plasma at indicated concentrations and then extracted and analyzed by HPLC. (B) Blood concentrations of HepIn-13 at indicated time points after tail vein injection of 1 mg of the compound. Data are the means of three independent experiments ±SD. (C) HepIn-13 is orally bioavailable. Analytical HPLC profiles (mAU at 220 nm) of blood plasma extracts: from tail vein injected with HepIn-13, untreated and oral gavage treated mice. (D) Mass Spectrometry confirmation of the presence of HepIn-13 in blood plasma in the peak highlighted by arrow in panel C.

To determine blood half-life of our compounds, we injected 1 mg of the compound into the tail vein and analyzed blood at different time points after injection. The compounds were extracted from blood plasma and analyzed by LCMS. To generate a standard curve, we added known concentrations of the compounds to mouse blood plasma and then extracted and analyzed them by LCMS (Figure 3, A). As expected, the blood concentration of the compounds dropped quickly immediately after the tail vein injections due to rapid tissue dissemination. The terminal blood half-life of HepIn-13 was calculated at 55 min, and similar values were obtained for other Hepsin inhibitors (Figures 3, B and S5).

To analyze the oral bioavailability, we delivered 3 mg of the test compounds by oral gavage and then analyzed blood for the appearance of the compounds 30 min after the treatment. We found that only one compound, HepIn-13, was detectable in blood after oral gavage (Figures 3, C-D and S6). Thus, we focused our further studies on HepIn-13.

### Molecular modeling of interactions between Hepsin and HepIn-13

**Figure 4:**
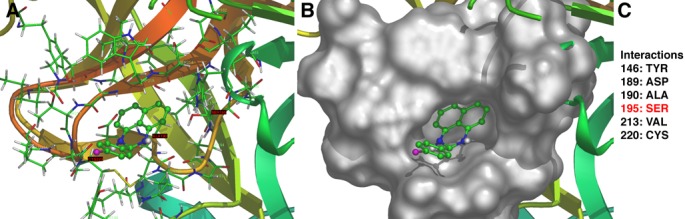
Predicted model of Hepsin–HepIn-13 interaction The best-ranked calculated docking of HepIn-13 with Hepsin predicts an interaction of the compound with the Hepsin catalytic domain. (A) Ribbon model of Hepsin-HepIn-13 interaction. (B) Surface model of Hepsin-HepIn-13 interaction. (C) Amino acids of Hepsin involved in interaction. Note, Ser353(195) is a part of Hepsin catalytic triad [23]

The crystal structure of Hepsin has been previously determined [23]. We used *in silico* molecular docking approaches to model interactions between Hepsin and HepIn-13 (http://www.dockingserver.com/web). The entire extracellular region of Hepsin was used in these unsupervised docking experiments. Interestingly, the best-ranked calculated docking pose demonstrates an interaction of HepIn-13 with the catalytic pocket of Hepsin (Figure 4). Moreover, one of the amino acids of the Hepsin catalytic triad, Ser195 (Ser353 in full-length Hepsin), is predicted to interact with HepIn-13. Thus, *in silico* docking of Hepsin and HepIn-13 places HepIn-13 within the catalytic pocket of Hepsin and predicts an interactions with one of the amino acids of the Hepsin catalytic triad.

### In vivo efficacy of HepIn-13 in the mouse model of metastatic prostate cancer

To determine whether HepIn-13 can attenuate or prevent prostate cancer progression *in vivo*, we performed animal studies. For this purpose, we chose to use *LPB-Tag/PB-Hepsin* mice, which is the only existing genetic model of metastatic prostate cancer that expresses Hepsin and consistently shows bone metastasis, which is prevalent in human prostate cancer [12]. LPB-Tag/PB-Hepsin mice upregulate expression of SV40 large T antigen and Hepsin in the prostate epithelium and develop primary prostate cancer with metastatic lesions in approximately 55% of the animals [12]. *LPB-Tag/PB-Hepsin* mice are derived from *LPB-Tag* animals (12T-7f adenocarcinoma line), which express SV40 large T antigen, but do not express Hepsin [12, 24]. *LPB-Tag* mice present with primary prostate tumors, but do not develop metastasis. Thus, *LPB-Tag/PB-Hepsin* model of prostate cancer was ideal for our analysis of Hepsin inhibitors, because the functional significance of Hepsin expression in these mice was already well established.

Considering the characteristics of the *LPB-Tag/PB-Hepsin* model, we anticipated that we would need to treat animals with HepIn-13 for a relatively long duration, starting at 10 weeks of age, when the animals present with low-grade prostate tumors. The most non-invasive long-term delivery of an orally bioavailable small molecule compound involves mixing the test compound with animal water or food. Thus, we first analyzed whether we can detect HepIn-13 in the animal blood, after the mice are exposed to the soft rodent chow containing varying concentrations of HepIn-13. With our limit of blood HepIn-13 detection at 1 μM, we found that exposure of the animals to rodent chow containing 0.25% of HepIn-13 resulted in the presence of HepIn-13 in blood at concentrations that are significantly higher than its IC50 (Figure S7). We also analyzed blood of mice exposed for 5 weeks to the food containing 0.25% of HepIn-13 for signs of liver toxicity; however, the levels of AST and ALT were within the normal ranges in these animals indicating absence of general liver damage. The animals did not exhibit signs of distress in this chronic dosing experiment. Thus, we decided to perform animal trials with HepIn-13 mixed with rodent chow at 0.25% and 0.1%.

**Figure 5:**
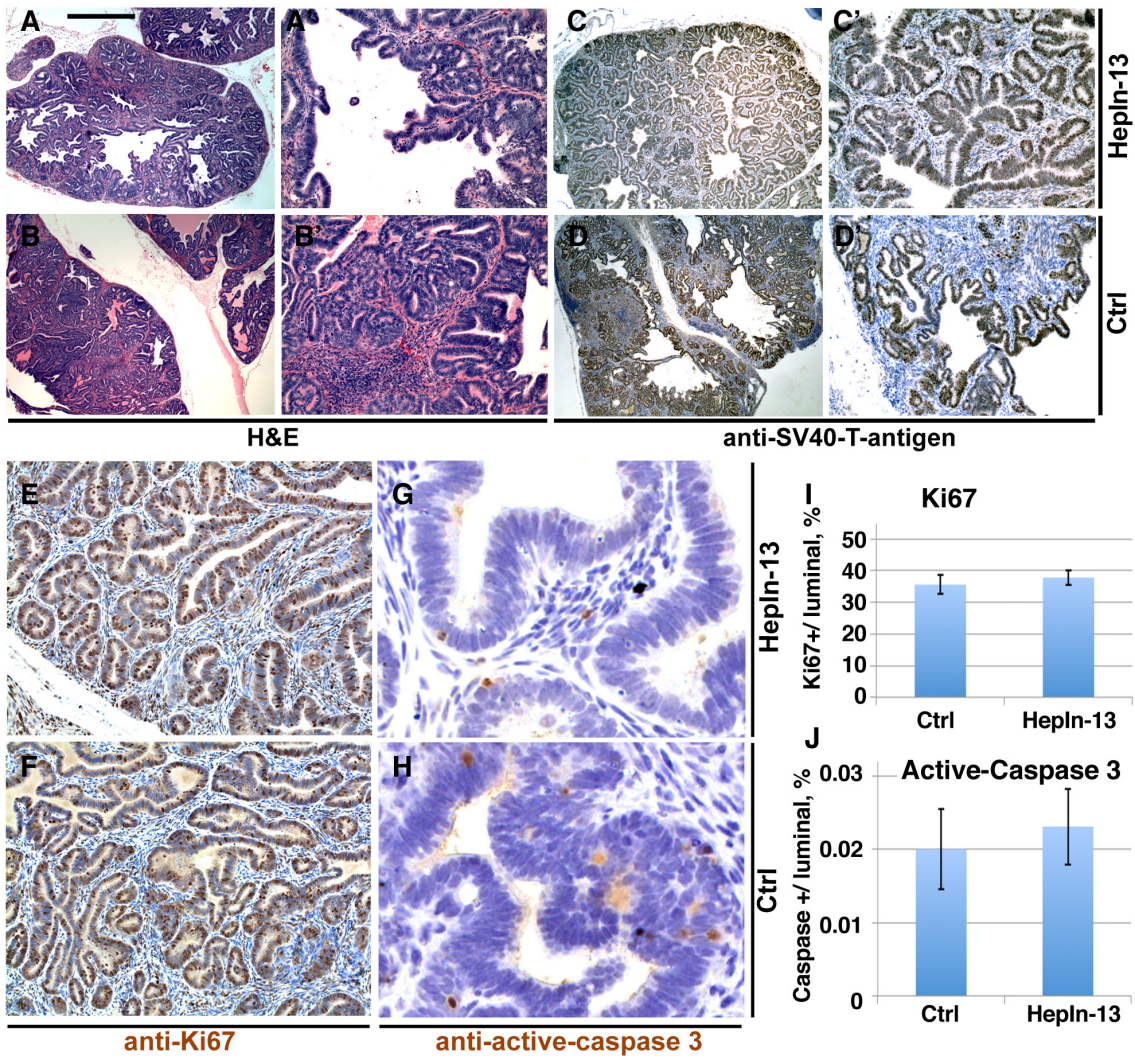
Primary prostate tumors in HepIn-13-treated (0.25%) and control LPB-Tag/PB-Hepsin mice (A-B') Hematoxylin and Eosin stainings of sections from primary prostate tumors in 23 week-old HepIn-13-treated (A, A') and untreated control (B, B') males. (C-D') Immunohistochemical staining of sections from primary prostate tumors in 23 week-old HepIn-13-treated (C, C') and control (D, D') males with anti-SV40 large T antigen. (E-F) Immunohistochemical staining of sections from primary prostate tumors in 23 week-old HepIn-13-treated (E, G) and control (F, H) males with anti-Ki67 (E, F) and anti-active-caspase 3 (G, H) antibodies. (I) Quantitation of anti-Ki67 staining. N=3. Bar graph shows mean values ±SD. (J) Quantitation of anti-active caspase 3 staining. N=3. Bar in A represents 0.5 mm in A, B, C, D, 100 μm in A', B', C', D', 55 μm in E, F, and 15 μm in G, H.

**Figure 6:**
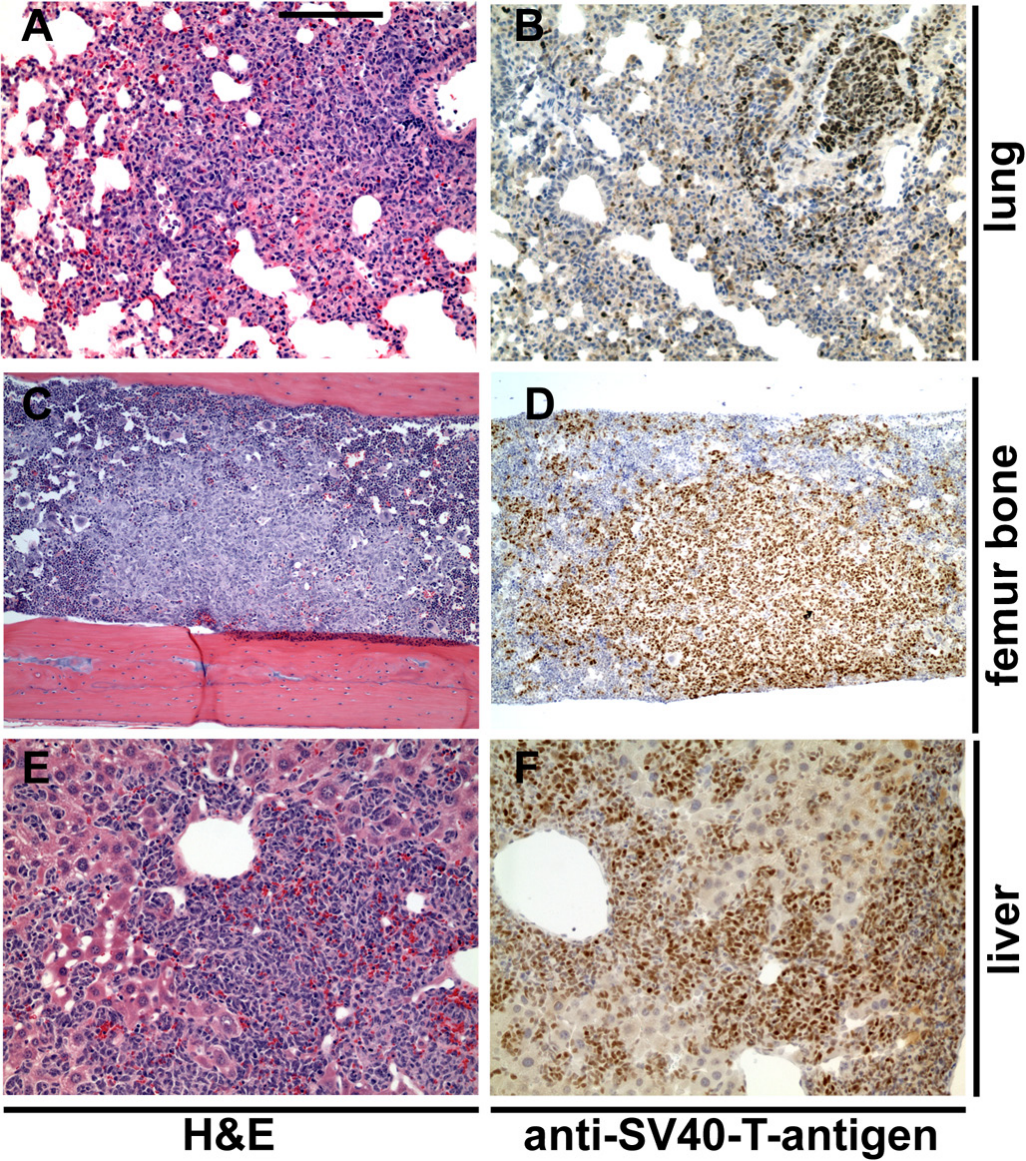
Metastatic lesions in control untreated *LPB-Tag/PB-Hepsin* mice. Hematoxylin and Eosin (A, C, E) and anti-SV40 large T antibody (brown in B, D, F) stainings of metastatic lesions in lung (A, B), bone (C, D) and liver (E, F) in 23 week-old *LPB-Tag/PB-Hepsin* males. Bar in A represents 200 μm in A, B, E, F, 360 μm in C, D.

For the animal trial, 10 week-old LPB-Tag/PB-Hepsin males were randomly assigned to one of 3 groups. One control group was exposed to soft rodent chow alone (12 mice), while the other two groups were exposed to the same rodent chow mixed with 0.1% (10 mice) or 0.25% (11 mice) of HepIn-13. Thirteen weeks later the animals were euthanized and their prostate glands, livers, lungs and bones were examined by histological staining and immunochistochemistry (Figures 5, 6). As expected, the control animal group presented with metastatic lesions in 66% of the animals, and bone metastasis in 42% of the animals (Table 1, Figure 6). While cellular proliferation, apoptotic cell death, blood vessel density (Figures 5, S8) and the size of the primary prostate tumors were not significantly changed in the group treated with 0.25% of Hepln-13 (mean tumor size 1.9±2.0 g in HepIn-13-treated versus 2.1±3.3 g in control animals), no metastasis was found in these mice (Table 1). The group exposed to 0.1% of HepIn-13 displayed intermediate metastasis phenotype (30% animals with metastasis, 20% bone metastasis). Overall, we conclude that HepIn-13 displays dose-dependent inhibition of Hepsin overexpression-relevant prostate cancer phenotypes in *LPB-Tag/PB-Hepsin* mice and blocks prostate cancer metastasis in this animal model.

**Table 1:** Incidence of metastasis and metastatic sites in control and HepIn-13-treated LPB-Tag/PB-Hepsin mice 10 week-old LPB-Tag/PB-Hepsin mice were randomly divided into 3 groups. Mice were exposed for 13 weeks to soft rodent chow alone (Control group), or chow mixed with 0.1% (HepIn-13 0.1% group) or 0.25% (HepIn-13 0.25% group) of HepIn-13 for 13 weeks. Animals were euthanized and their prostate glands, livers, lungs and bones were analyzed by histology and IHC staining with anti-SV40 T antibodies. The number of mice displaying indicated metastatic lesions relative to the total number of mice (in parentheses) is shown. * indicates P<0.05. ** indicates P<0.01. Two-tailed Fisher's exact test.

	Metastatic lesions			
	Total	Liver	Lung	Bone
Control group	8(12)	8 (12)	4 (12)	5 (12)
HepIn-13 (0.1%) group	3(10)	3 (10)	1 (10)	2 (10)
HepIn-13 (0.25%) group	0(11)**	0 (11)**	0 (11)	0 (11)*

## DISCUSSION

We show here that HepIn-13 inhibits Hepsin and blocks prostate cancer metastasis. Importantly, HepIn-13 was able to block metastasis to the bone, which is the most common site of metastasis in human prostate cancer. Interestingly, despite the high penetrance of bone metastasis in human prostate cancer, this has been difficult to replicate in mouse models. Consistent bone metastasis was reported in mice with prostate cancer caused by deletion of *PTEN/P53* and the loss and subsequent re-activation of Telomerase [25]; however, subsequent pathological analysis suggested direct bone invasion from large primary tumors rather than *bona fide* bone metastasis [26]. LPB-Tag/PB-Hepsin mice is a genetic model of prostate cancer which consistently develops bone metastasis [12, 26]. We have previously reported that 20% of 21 week-old LPB-Tag/PB-Hepsin mice present with femur bone lesions. In this study we used a different animal feed (soft transgenic dough) and found that by 23 weeks of age 42% of LPB-Tag/PB-Hepsin mice present with bone metastasis. All the mice with lesions in femur bones also displayed lesions in spine, indicating broad skeletal involvement in affected individuals. Since spine lesions may be the result of a direct primary tumor invasion, we believe that the presence of femur lesions in LPB-Tag/PB-Hepsin mice is more significant, because it indicates *bona fide* bone metastasis. Treatment of LPB-Tag/PB-Hepsin mice with Hepsin inhibitor HepIn-13 completely suppressed the development of bone metastasis, strongly suggesting that Hepsin plays an important role in the skeletal spread of prostate cancer.

The analysis of HepIn-13 in a single autochthonous model of prostate cancer is an important limitation of this study. Additional Hepsin overexpressing models of prostate cancer will need to be developed to analyze the efficacy of HepIn-13. We have crossed *PB-Hepsin* [12] and *Pr-Cre4/PTEN^fl/fl^* [27] mice, hypothesizing that overexpression of Hepsin in *PTEN^−/−^* prostate epithelial cells could promote bone metastasis. However, we found that the synthetic Probasin (PB) promoter that was used in the generation of PB-Hepsin mice [28] is inactivated in *PTEN^−/−^* cells *in vivo*. Therefore, different approach to drive Hepsin expression will have to be used in the future to generate new models to analyze the efficacy of HepIn-13 in inhibition of prostate cancer bone metastasis.

While HepIn-13 is the first small molecule Hepsin inhibitor used in an animal model of prostate cancer, a protein-based Hepsin inhibitor, Kunitz domain-1, was previously used in a xenograft model of prostate cancer [18]. In that model, LnCaP-34 cells displayed a Hepsin-dependent ability to invade and develop lymph node metastasis [18]. Treatment of mice carrying LnCaP-34 tumors with Kunitz domain-1 decreased contralateral prostate invasion (46% weight reduction) and lymph node metastasis (50% inhibition) [18].

In addition to Kunitz domain-1, development of several antibodies specifically inhibiting Hepsin have been reported [13, 29, 30]. While Hepsin antibodies are likely to be useful for prostate cancer imaging, the therapeutic use of these antibodies for tumor eradication may be somewhat limited due to the prominent expression of endogenous Hepsin in hepatocytes [31]. *Hepsin* is not an essential gene and *Hepsin* knockout mice are viable and fertile [32, 33]; however, these animals exhibit hearing loss due to developmental deformities in the cochlea [34]. Moreover, *Hepsin* knockout mice display enlarged hepatocytes and narrowed liver sinusoids [35]. Consistent with the phenotype of *Hepsin* knockout mice, we did not observe any prominent deficiency in mice treated with Hepsin inhibitors; however, more careful examination will be necessary in the future.

One of possible limitation of HepIn-13 is its potential inhibitory effect on other proteases. While we found that HepIn-13 did not inhibit Matriptase, it is always possible that HepIn-13 can inhibit other enzymes. Considering the lack of toxicity associated with long-term HepIn-13 exposure in mice, it appears unlikely that HepIn-13 inhibited essential enzymes. Therefore, the potential off-target activity of HepIn-13 is unlikely to pose a significant problem.

While we found that HepIn-13 was orally bioavailable, we used relatively high concentration of HepIn-13 (0.25%) in animal food to achieve detectable levels in blood. Therefore, it appears that the oral bioavailability of HepIn-13 is relatively poor and additional modifications will be necessary to enhance the drug properties without decreasing the potency and specificity.

## METHODS

### Reagents

Small molecule compounds were either purchased from ChemBridge Corporation or were synthesized (HepIn-13). HepIn-13 was prepared by heating 1,8-diaminonaphthalene (1 mM) and 4-bromobenzaldehyde (1 mM) overnight in absolute ethanol. The resulting mixture was concentrated and the resulting dark brown/purple solid was recrystallized twice from 95% ethanol to yield a light purple crystalline solid (64% yield). The product was homogeneous by HPLC (>95% purity) and had ^1^H NMR and mass spectra consistent with the proposed structure. The chromogenic peptide pyroGlu-Pro-Arg-pNA (s-2366) was purchased from DiaPharma. Rabbit anti-human Hepsin polyclonal antibody was purchased from Cayman Chemical (#100022). Anti-Flag antibody was purchased from Sigma (F1804). Anti-HA antibody was purchased from Roche (#1867423). Goat anti-rabbit secondary antibody was purchased from Jackson ImmunoResearch. Human recombinant Matriptase was purchased from R&D Systems. Molecular biology-grade DMSO was purchased from Sigma. Purified recombinant human pro-HGF was obtained from Dr. George F. Vande Woude (Van Andel Research Institute).

### Recombinant Hepsin expression purification and in vitro activity assays

Extracellular part of human Hepsin (amino acids 45-417) containing the scavenger receptor cysteine-rich (SRCR) and trypsin-like serine protease domains was expressed and purified from S2 cells using the Drosophila Inducible/Secreted Expression System (Invitrogen), as previously described [36]. The Hepsin V_max_, K_m_ and Hepsin inhibition were determined using chromogenic substrate pyroGlu-Pro-Arg-pNA as described [20]. Briefly, the inhibition assays were performed by incubating the compounds with Hepsin in the assay buffer (30mM Tris-HCl, pH8.4; 30mM imidazole, 200mM NaCl and 1% DMSO) for 30 minutes at room temperature followed by addition of the substrate at the observed Km and incubation for 3h. The endpoint absorbance was measured using a VersaMax microplate reader (Molecular Devices). Residual Hepsin activity was observed relative to DMSO controls. IC_50_ was calculated by fitting the data to a four-variable nonlinear regression using GraphPad Prism5.

### Recombinant DNA constructs

For generation of a Doxycycline- regulated Hepsin expression plasmid, full-length human Hepsin, retaining the last intron for transcript stabilization, was amplified by stitching PCR using both cDNA and genomic DNA templates. The resulting fragment was cloned into EcoRI/SalI sites of pTRE-Tight (Clontech). For generation of HA-tagged pro-HGF expression plasmid, mouse cDNA library was PCR amplified with oligos containing the restriction enzyme cutting sites and C-terminal HA sequence. The fragment was cloned into SacI/SalI sites of pIRES-hrGFP-2a (Stratagene). All DNA constructs were sequence verified.

### Cell lines and cell culture

LNCaP, CHO, PC3 and HEK293FT cells were obtained from ATCC. LNCaP and PC3 cells were maintained in RPMI1640 supplemented with 10% FBS and penicillin/streptomycin. HEK293FT cells were maintained in DMEM supplemented with 10% FBS, nonessential amino acids and penicillin/streptomycin. CHO cells were grown in F-12 media supplemented with 10% FBS and penicillin/streptomycin. Drosophila S2 cells expressing inducible secreted extracellular part of human Hepsin were grown in Schneider's medium (Invitrogen) containing 10% fetal bovine serum, and penicillin/streptomycin [21]. TET-OFF HEK293 cells were purchased from Clontech (C3008-1). For generation of a cell line expressing full-length human Hepsin (HEK293-Hepsin), TET-OFF HEK293 cells were stably transduced with a mixture of pTRE-Tight-hHepsin and pBabe and stable clones were selected with puromycin. For generation of a cell line expressing secreted HA-tagged full-length mouse pro-HGF (HEK293-pro-HGF-HA), HEK293 cells were stably transduced with a mixture of pIRES-hrGFP-2a (Stratagene) and pBabe and stable clones were selected with puromycin.

### Cell-based Hepsin activity assay

HEK293 cells stably transduced with full-length human Hepsin were used in cell-based Hepsin activity assays. To obtain HA-tagged pro-HGF substrate, HEK293 cells stably transduced with HA-tagged pro-HGF were washed with PBS and incubated in serum-free media for 8 hours. The conditioned media containing HA-tagged pro-HGF was collected, filtered through 0.2 μm surfactant-free cellulose acetate filter, concentrated by ultrafiltration (Amicon Ultra, Millipore) and kept in aliquots at -80°C. For the activity assay, the HEK293-Hepsin cells were plated in a 24-well plate at 200,000 cells/well. Forty-eight hrs later, cells were rinsed with serum-free media and cultured for 4 hours in serum-free media with 1% DMSO or indicated test compounds dissolved in DMSO. The conditioned serum-free media containing HA-tagged pro-HGF was added and the cells were cultured for additional 90 minutes. The resulting extracellular media was collected and the cleavage of pro-HGF was analyzed using western blotting with anti-HA antibodies.

### *In vivo* efficacy trial in a genetic animal model of metastatic prostate cancer

*LPB-Tag/PB-Hepsin* mice express SV40 large T antigen and Hepsin transgenes in luminal prostate epithelium and develop prominent primary prostate cancer with metastatic lesions in liver, lung, and bone [12]. *LPB-Tag/PB-Hepsin* males were generated as F1 in the crossing of *LPB-Tag* transgenic females (12T-7f adenocarcinoma line, CD-1 background) [24] with *PB-Hepsin* transgenic males (C57BL/6J background), as described [12].

For the *in vivo* experiment, the 10-week-old LPB-Tag/PB-Hepsin males were randomly divided into 3 groups. The control group was exposed to soft Transgenic Dough Diet (Bio-Serv, S3472) with 10% of powdered rodent chow and two experimental groups were exposed to the same food mixed with either 0.1% or 0.25% of the compound HepIn-13. After 13 weeks of exposure, the animals were euthanized and their prostates, livers, lungs, and spine and femur bones were analyzed.

### Determination of compound pharmacokinetics in mice

CD-1 mice (Charles River) were used in general pharmacokinetics experiments. All experiments involving mice were conducted in compliance with Fred Hutchinson Cancer Research Center Committees on Use and Care of Animals guidelines. The indicated compounds were solubilized in 1:1 Ethanol:Cremophore EL (Merck) mixture and diluted with 9 volumes of PBS. To determine the highest dose resulting in acute toxicity, escalating doses of the compounds were injected into the tail vein of adult mice and animals were monitored for 2 days. No signs of acute toxicity were observed in these experiments for all investigated compounds injected at the maximum practical dose of 20mg/kg. To determine the blood half-life, 1 mg of the indicated compound was injected into the tail vein of adult mice and the blood was collected at different time points after injection. The compounds were extracted from blood plasma with ethyl acetate, air dried, resuspended in Acetonitrile and analyzed by HPLC (Agilent Technologies, ACE 3 C8-300, 150 x 3.0mm, ACE-212-1503, A 72063). The HPLC peaks were subsequently analyzed using Mass Spectrometry (LTQ-OrbiTrap, Agilent). To test oral bioavailability, 3 mg of the indicated compound was delivered by oral gavage and blood was drawn at different time points after the treatment and analyzed for the presence of the compounds as described above. Similar approach was used to test for the presence of the indicated compounds in blood of the animals exposed to Transgenic Dough Diet (Bio-Serv, S3472) with 10% powdered rodent chow and 0.1% or 0.25% of the test compound. Levels of Aspartate transaminase and Alanine aminotransferase (AST and ALT) in blood were measured by Phoenix Central Laboratory (Mukilteo, WA).

### Western blotting

Total prostate protein lysates were generated by manual grinding of frozen in liquid nitrogen prostates using prechilled on dry ice Mortar and Pestle. The resulting frozen powder was extracted with the protein lysis buffer containing: 1xPBS, 1% SDS, 1% NP40, 2% Tween 20, and 1.5 M Urea. The proteins were separated on 4-12% NuPAGE gels and transferred to PVDF membrane using the iBlot system (Invitrogen). The membrane was blocked in TBST buffer containing: 5% non-fat milk, 2% normal goat serum in 50 mM Tris-HCl, pH 8.0, 100 mM NaCl, 0.1% Tween-20 and incubated overnight at +4°C in the same buffer containing anti-Hepsin (Cayman, 1:1000), anti-HA (Roche, 1:1000) or anti--actin (Sigma A5441, 1:20,000), antibodies. The relevant HRP-labeled secondary antibodies (1:5000) were purchased from Jackson ImmunoReserch Laboratories and the blots were developed using ECL (Pierce). Expression levels were quantified by densitometry using ImageQuantTL.

### Immunohistochemistry

Tissue samples were processed, embedded in paraffin, and sectioned at 5μm. Sections were deparafinized, rehydrated, and processed for immunostaining with anti-SV40-Tag antibody (Millipore, DP02). The ABC MOM kit (Vector Laboratories) was used for immunohistorchemistry. Antibodies were detected using DAB peroxidase substrate kit and sections were counterstained with hematoxylin QS (both from Vector Laboratories).

### Molecular Docking

To model the potential interaction between Hepsin and identified in this study Hepsin inhibitors, molecular docking of Hepsin and the identified small molecule-inhibitors was performed by using DockingServer [37] (www.dockingserver.com). The previously determined crystal structure of extracellular region of human Hepsin [23] and 3-dimentional structure of HepIn-13 were used in these experiments. Docking calculations were then performed by using AutoDock 4 [38] integrated in DockingServer. Docking simulations were carried out by using the Lamarckian Genetic Algorithm. The initial position, orientation, and torsions of the inhibitor molecules were set randomly, and all rotatable torsions were released during docking. Each docking experiment was derived from 100 different runs that were set to terminate after a maximum of 2500000 energy evaluations. The conformer with the lowest docking energy calculated with AutoDock 4 scoring function was selected as the final binding conformation.

### Statistical Analysis

For all graphs, data presented are mean value ± SD. Statistics were calculated using GraphPad Prism 6. Statistical significance was determined by the unpaired Student's t test, the two-tailed Fisher's exact test (for comparison of incidence of metastasis) and Mann–Whitney test (for comparison of tumor size). P value is indicated by asterisk(s) in the figures. *, denotes P<0.05; **, P<0.01. Differences at P<0.05 and lower were considered statistically significant.

## SUPPLEMENTARY FIGURES AND REFERENCES


