# Supplementary material for: Targeted inhibition of cell-surface serine protease Hepsin blocks prostate cancer bone metastasis
Source: Oncotarget. 2014 Mar 16;5(5):1352–62. doi: 10.18632/oncotarget.1817 (PMC4012739; doi:10.18632/oncotarget.1817)
Supplement: Supplementary file 1 [file oncotarget-05-1352-s001.pdf]

Supplementary Materials

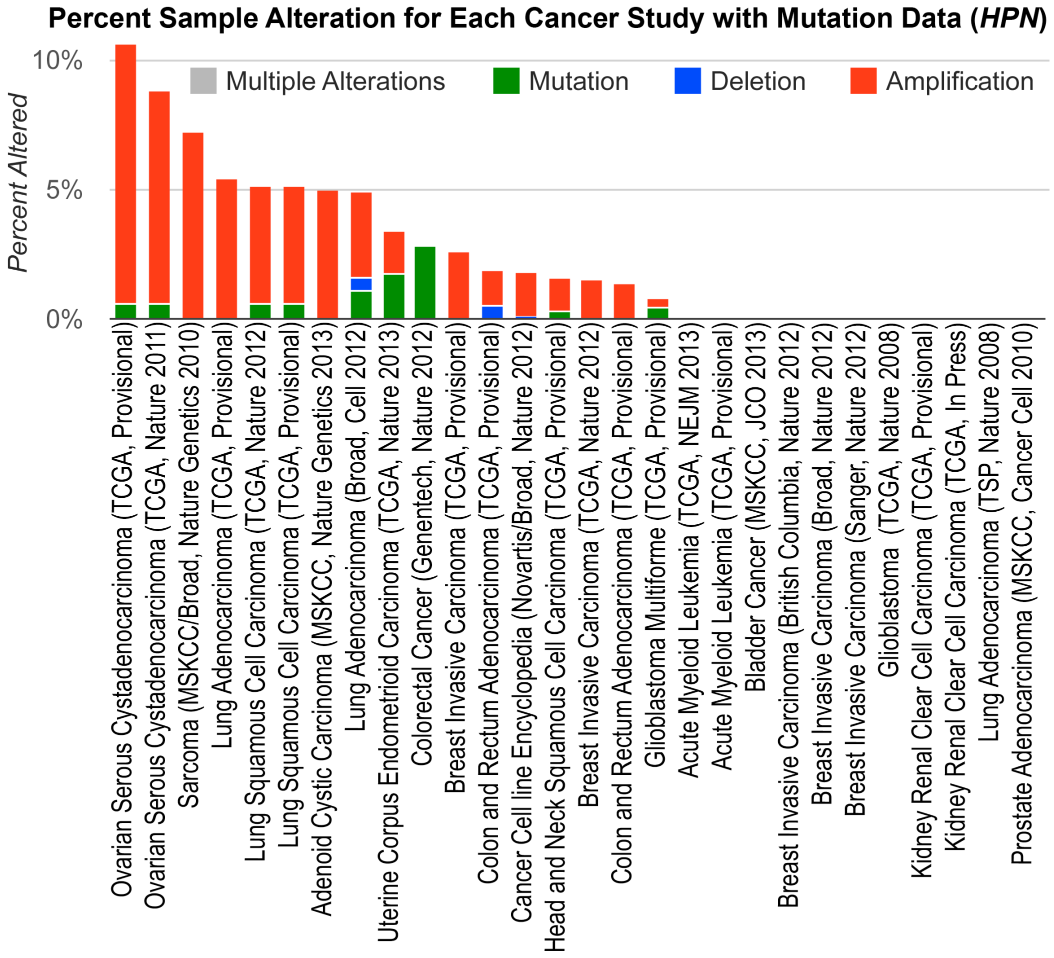

Figure S1. Summary of *Hepsin* (*HPN*) gene mutations, deletions and amplifications in multiple cancer types. cBio analysis (http://www.cbioportal.org/public-portal/) (Cerami et al., 2012; Gao et al., 2013).

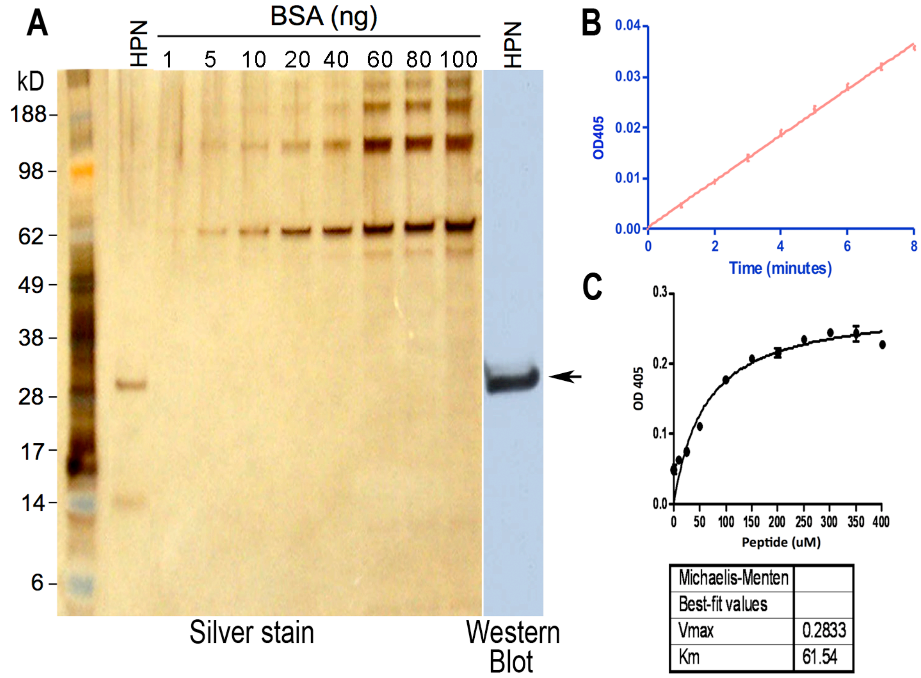

Figure S2. Characterization of recombinant purified Hepsin. **(A)** Silver stained SDS-PAGE gel and Western Blot (anti-Hepsin catalytic domain) analysis of chromatographically purified Hepsin, which was produced as a recombinant protein in Drosophila S2 cells. **(B)** In vitro Hepsin activity assay. The chromogenic serine protease substrate pyroGlu-Pro-Arg-pNA was added to purified Hepsin and enzyme activity was observed as a linear increase in absorbance at 405 nm over time. C, Vmax and Km determination. Substrate-velocity data fit with nonlinear regression using Graphpad Prism5 was used to calculate Vmax and Km.

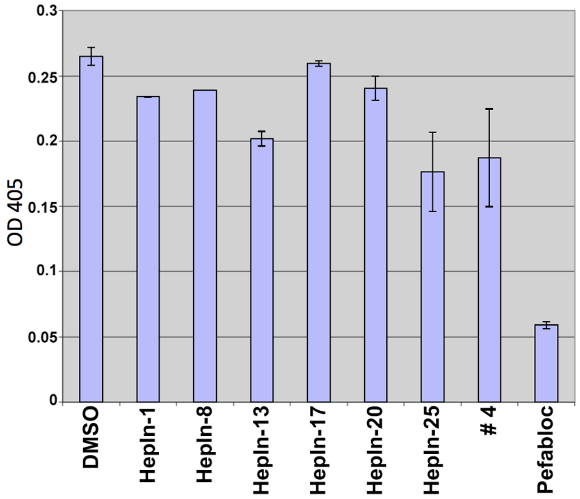

Figure S3. Minimal impact on Matriptase activity by Hepsin inhibitor #4 (Chevillet et al., 2008) and its derivatives. Purified recombinant Matriptase was preincubated with 10 µM of indicated compounds for 30 min. The residual percent activity of the enzyme toward the chromogenic substrate was determined with microplate reader at 405 nm. Data are the means of three independent experiments ±SD.

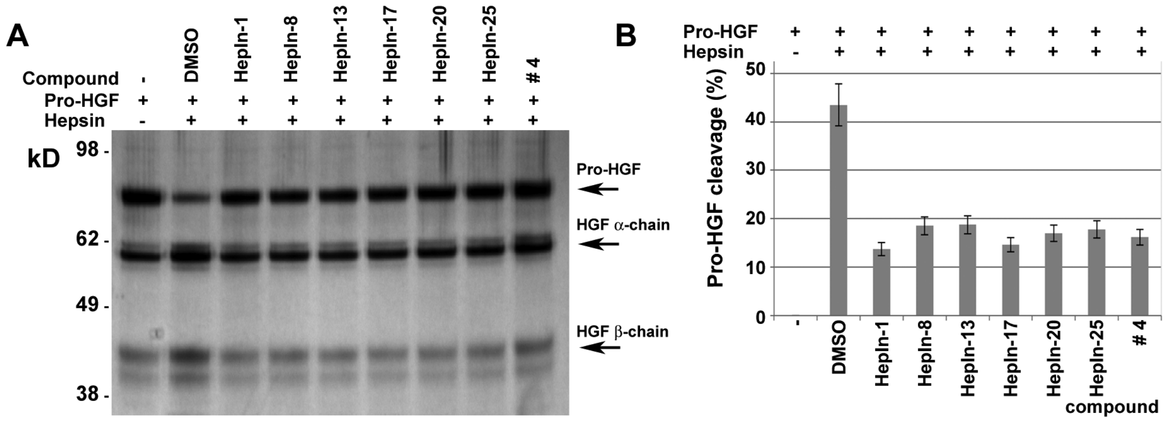

Figure S4. Inhibition of Hepsin activity toward pro-HGF. Purified Hepsin was preincubated with 10 μM of the indicated compounds and exposed to purified pro-HGF. **(A)** Silver stained SDS-PAGE. **(B)** Quantitation. Data are the means of three independent experiments ±SD.

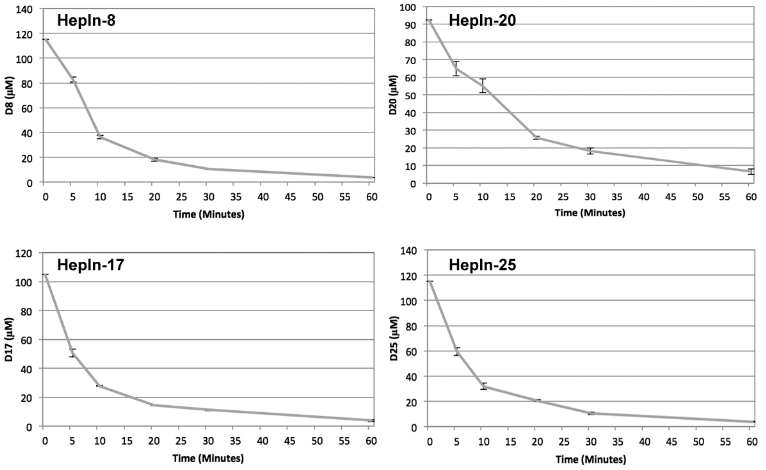

Figure S5. Blood concentrations of HepIn-8, HepIn-17, HepIn-20 and HepIn-25 at indicated time points after tail vein injection of 1 mg of the compound. Data are the means of three independent experiments ±SD.

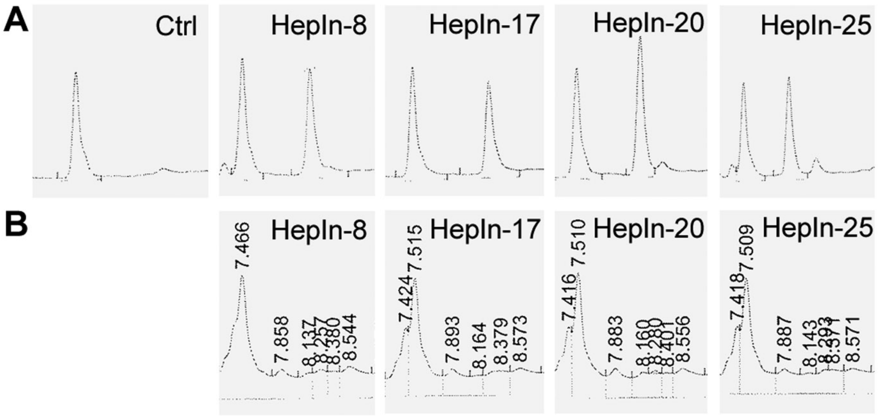

Figure S6. (A) Analytical HPLC profiles (mAU at 220 nm) of blood plasma extracts from mice injected intravenously with empty vehicle control (Ctrl), HepIn-8, HepIn-17, HepIn-20 and HepIn-25. **(B)** Analytical HPLC profiles (mAU at 220 nm) of blood plasma extracts from mice treated by oral gavage with HepIn-8, HepIn-17, HepIn-20 and HepIn-25.

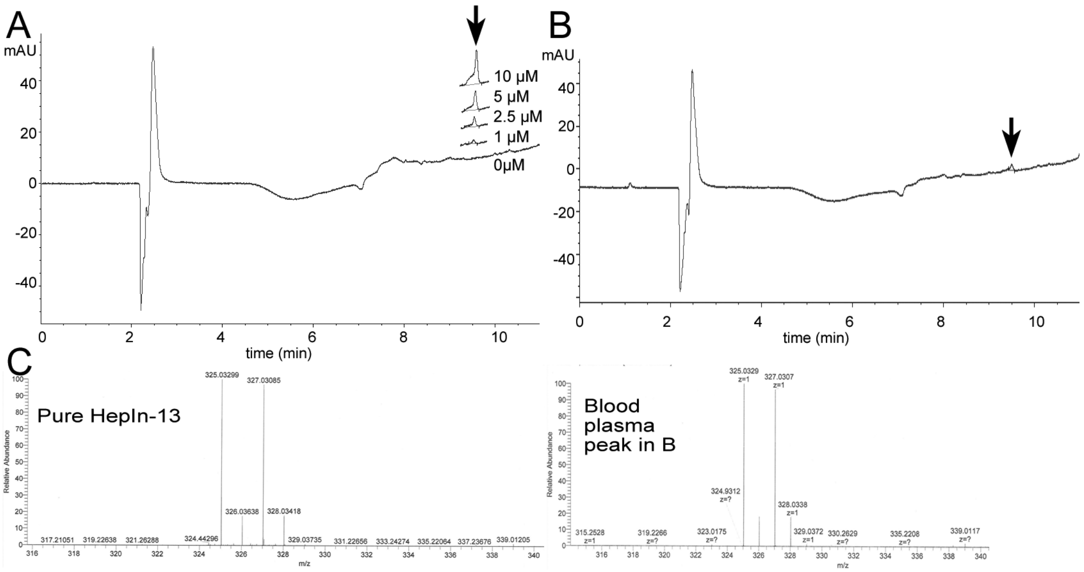

Figure S7. HepIn-13 in blood plasma after exposure to food containing HepIn-13. **(A)** Analytical HPLC profiles (mAU at 254 nm) of HepIn-13 which was dissolved in the mouse blood plasma at indicated concentrations and then extracted and analyzed by LCMS. **(B)** Analytical HPLC profile (mAU at 254 nm) of compounds extracted from blood plasma of the animal exposed to food containing 0.25% of HepIn-13. C, Mass Spectrometry confirmation of the presence of HepIn-13 in blood plasma in the peak highlighted by arrow in panel B.

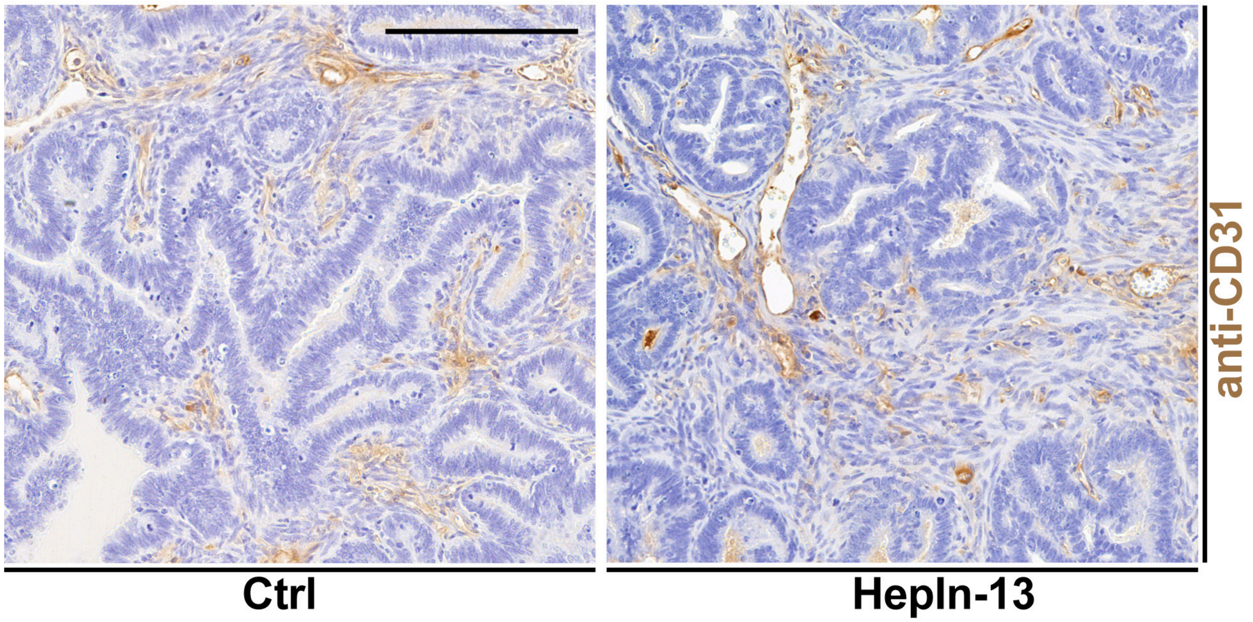

Figure S8. Immunohistochemical staining of sections from primary prostate tumors in 23 week-old control (Ctrl) and HepIn-13-treated (HepIn-13, 0.25%) mice with anti-CD31 antibodies. Bar represents 48 μm.

Supplementary References

Cerami, E., J. Gao, U. Dogrusoz, B.E. Gross, S.O. Sumer, B.A. Aksoy, A. Jacobsen, C.J. Byrne, M.L. Heuer, E. Larsson, Y. Antipin, B. Reva, A.P. Goldberg, C. Sander, and N. Schultz. 2012. The cBio cancer genomics portal: an open platform for exploring multidimensional cancer genomics data. *Cancer discovery* 2:401-404.

Chevillet, J.R., G.J. Park, A. Bedalov, J.A. Simon, and V.I. Vasioukhin. 2008. Identification and characterization of small-molecule inhibitors of hepsin. *Mol Cancer Ther* 7:3343-3351.

Gao, J., B.A. Aksoy, U. Dogrusoz, G. Dresdner, B. Gross, S.O. Sumer, Y. Sun, A. Jacobsen, R. Sinha, E. Larsson, E. Cerami, C. Sander, and N. Schultz. 2013. Integrative analysis of complex cancer genomics and clinical profiles using the cBioPortal. *Sci Signal* 6:pl1.
